# Symptoms and signs of urogenital cancer in primary care

**DOI:** 10.1186/s12875-023-02063-z

**Published:** 2023-04-26

**Authors:** Knut Holtedahl, Lars Borgquist, Gé A. Donker, Frank Buntinx, David Weller, Christine Campbell, Jörgen Månsson, Victoria Hammersley, Tonje Braaten, Ranjan Parajuli

**Affiliations:** 1 Department of Community Medicine, UiT The Arctic University of Norway, 9037 Tromsø, Norway; 2 Department of Health, Medicine and Caring Sciences, Linköping University, 58183 Linköping, Sweden; 3 Primary Care Database, Netherlands Institute for Health Services Research, Otterstraat 118, Utrecht, 3513 The Netherlands; 4 Department of General Practice, KU Leuven, Oude Markt 13, 3000 Leuven, Belgium; 5 Maastricht University, P.O. Box 616, Maastricht, 6200 The Netherlands; 6 Usher Institute of Population Health Sciences and Medical Informatics, University of Edinburgh, Edinburgh, EH8 9AG UK; 7 Department of Public Health and Community Medicine/Primary Health Care, University of Gothenburg, Box 100, 40530 Gothenburgh, Sweden; 8 Independent researcher, Tromsø, Norway

**Keywords:** Neoplasms, Urogenital cancer, Bladder cancer, Renal cancer, Prostate cancer, Cervical cancer, Uterine body cancer, Ovarian cancer, General practice, Primary health care

## Abstract

**Background:**

Urogenital cancers are common, accounting for approximately 20% of cancer incidence globally. Cancers belonging to the same organ system often present with similar symptoms, making initial management challenging. In this study, 511 cases of cancer were recorded after the date of consultation among 61,802 randomly selected patients presenting in primary care in six European countries: a subgroup analysis of urogenital cancers was carried out in order to study variation in symptom presentation.

**Methods:**

Initial data capture was by completion of standardised forms containing closed questions about symptoms recorded during the consultation. The general practitioner (GP) provided follow-up data after diagnosis, based on medical record data made after the consultation. GPs also provided free text comments about the diagnostic procedure for individual patients.

**Results:**

The most common symptoms were mainly associated with one or two specific types of cancer: ‘Macroscopic haematuria’ with bladder or renal cancer (combined sensitivity 28.3%), ‘Increased urinary frequency’ with bladder (sensitivity 13.3%) or prostatic (sensitivity 32.1%) cancer, or to uterine body (sensitivity 14.3%) cancer, ‘Unexpected genital bleeding’ with uterine cancer (cervix, sensitivity 20.0%, uterine body, sensitivity 71.4%). ‘Distended abdomen, bloating’ had sensitivity 62.5% (based on eight cases of ovarian cancer). In ovarian cancer, increased abdominal circumference and a palpable tumour also were important diagnostic elements. Specificity for ‘Macroscopic haematuria’ was 99.8% (99.7–99.8). PPV > 3% was noted for ‘Macroscopic haematuria’ and bladder or renal cancer combined, for bladder cancer in male patients. In males aged 55–74, PPV = 7.1% for ‘Macroscopic haematuria’ and bladder cancer. Abdominal pain was an infrequent symptom in urogenital cancers.

**Conclusions:**

Most types of urogenital cancer present with rather specific symptoms. If the GP considers ovarian cancer, increased abdominal circumference should be actively determined. Several cases were clarified through the GP’s clinical examination, or laboratory investigations.

## Background

In a previous study, we have analysed the frequency of abdominal symptoms in general practice consultations, comparing patients with and without a subsequent cancer diagnosis [1, 2]. The subgroup of patients with colorectal cancer was further analysed according to symptoms and pathways to diagnosis [3].

The present article describes the subgroup of patients diagnosed with urogenital cancer in our cohort.

Globally, one in five cases of new cancer is a urogenital cancer, and they account for about one in seven cancer deaths [4]. UK referral guidelines (NICE) recommend that general practitioners (GPs) refer patients when the positive predictive value (PPV) of symptoms exceeds 3% [5]. However, primary care data for urogenital cancer is scarce. NICE guidelines for bladder [6] or prostate [7] cancer are not specifically aimed at GPs. This is a challenge for primary care.

A recent publication from GLOBOCAN data reported prostate cancer as the second most common cancer and the fifth leading cause of cancer deaths among men worldwide [8]. Abdominal symptoms in prostate cancer are ambiguous. However, lower urinary tract symptoms such as nycturia, erectile dysfunction and hematuria have been related to prostate cancer diagnosis in primary care [9]. Renal and bladder cancers account for approximately 2% and 3% of global cancer diagnoses, respectively [8]. There are no screening programmes for these cancer types; thus, the diagnosis is based mainly on investigations of presenting symptoms. Macroscopic haematuria is reported as the most common predictor for both renal and bladder cancers [10]. Further investigation of haematuria for renal and bladder cancer is a well-established procedure in primary care. One recent systematic review examining the association between abdominal symptoms and bladder or renal cancer in primary care reported haematuria as a stronger predictor of cancer among males, with a positive predictive value (PPV) almost twofold compared to females. Furthermore, the review stated that recurrent urinary tract infection combined with haematuria in older patients may be a typical feature of bladder cancer [11].

Cervical cancer is the eighth most frequently diagnosed as well as the ninth leading cause of cancer deaths worldwide. It is considered highly preventable because of the effective primary and secondary prevention measures that are Human papillomavirus (HPV) vaccine and screening programme, respectively.

Postmenopausal vaginal bleeding is considered to be the most common symptom for uterine cancer [12]. Abdominal pain and distension can present as vague symptoms for both ovarian and uterine cancers, however women over 50 with these symptoms must be assessed thoroughly.

Diagnostic delays in urogenital cancers can occur as in other cancer types [13]. Patient intervals in the diagnosis of renal and bladder cancer were reported to be brief, and most of the delay happened in the referral facilities in a study of diagnostic pathways [11]. Early diagnosis of symptomatic urogenital cancers certainly improves patient outcomes and survival as they are highly treatable in the early stage [13].

Non-specific symptoms have previously been shown to have low cancer relevance in themselves, but they increase in importance when associated with an abdominal symptom [14]. Hence, we aimed at analyzing both non-specific and alarm abdominal symptoms in primary care among patients with urogenital cancer in the present study.

## Methods

### Initial registrations

For a detailed description of the methods, see [5]. Between 25 February 2011 and 27 July 2011, GPs recruited through The Cancer and Primary Care Research International Network (Ca-PRI), registered 67,809 consecutive consultations with 61,802 patients 16 years and older in Norway, Denmark, Sweden, the Netherlands, Belgium and Scotland. For initial registrations, participating GPs received a desktop workbook containing daily registration sheets, one for each of ten working days (the form can be viewed in the UiT Open Research Data repository), plus instructions about how to record abdominal symptoms. For patients with such symptoms, more general, non-specific symptoms and further diagnostic action were also recorded. For patients with more than one consultation within the ten-day period, the last consultation was used as the reference date of consultation. Symptoms recorded during different consultations were all included, with the longest duration noted.

Abdominal and general symptoms listed had been selected based on medical literature related on cancer symptomatology. Researchers were blinded for any person identifying characteristics.

### Follow-up

Eight months after each GP’s consultation period, GPs who had completed the initial registration sheets received forms for recording details of patients diagnosed with a new or recurring cancer after the consultation date (the form can be viewed in the UiT Open Research Data repository). The GPs were given their individual consultation dates and used their electronic records to identify these patients. The form was a simplified and revised version of a form used in two previous studies [15, 16]. All GPs were asked to supply anonymous information about the patients diagnosed with cancer during the follow-up period, whether they had presented symptoms during the initial survey or not. Free text comments accompanied multiple choice information about the diagnostic process, especially the role of clinical examination, laboratory tests ordered by the GP, and diagnostic procedures (typically outside the surgery). Further symptoms, described in the medical record and originating between the consultation date and the date of diagnosis, were mainly reported in the GPs’ free text comments. They were asked: “Write in short form what primarily made you (or another physician) suspect cancer in this particular patient”. Most free-text descriptions enabled recoding of ‘After consultation’ symptoms into one of the pre-registered symptoms used in the original registration forms. Two reminders were sent to GPs. From a total of 640 patients diagnosed with cancer, 129 patients were excluded from the study due to previously known, stable or progressive cancer (*n* = 69), misdiagnosis (*n* = 4), precancerous or basal cell carcinoma (*n* = 31), or missing information on whether the cancer was new, recurrent or prevalent (*n* = 25).

### Statistics

Statistical analyses were performed using SPSS, version 22. For each combination of symptoms and cancer sites considered, sensitivity was calculated as the proportion presenting the symptom among the patients diagnosed with cancer. Specificity was calculated as the proportion not presenting the symptom among the cancer-free patients. The positive predictive value was calculated as the proportion diagnosed with cancer among the patients with symptoms.

The 95% confidence intervals for sensitivity, specificity and PPV were computed using the Wilson method.

We used the STARD checklist when generating study output [17].

## Results

Completed questionnaires were received from 493 GPs, and 315 (64%) also returned follow-up forms for one or more subsequent cancer patients. Abdominal symptoms were recorded in 6264 patients (10.1%). Among the cancer-free patients, 143 presented macroscopic haematuria, 737 presented increased urinary frequency, and 195 patients presented unexpected genital bleeding.

After exclusion of 129 cancer patients, 511 patients with new or recurrent cancer were included. Of those there were 134 (26,2%) patients with the six most common urogenital types of cancer, in the following organs: Bladder/ureter/urethra, kidney, prostate, cervix uteri, corpus uteri, ovary. 114 were new incidences and 20 were recurrences. Prostate cancer was the most frequent (Table 1). There was only one case of testicular cancer (excluded from the analyses). The majority of bladder and renal cancer were in males, at similar rates to the Norwegian cancer registry Figs. [18].

**Table 1:** Number of patients with abdominal symptoms recorded during consultation, and between consultation and diagnosis of all cancers (*n* = 511) and urological cancer (*n* = 134)

	**All cancer (*N* = 511)**	**Urogenital cancer (N = 134)**	**Bladder (*N* = 30)**		**Renal (*N* = 16)**		**Prostate (*N* = 56)**		**Cervix uteri (*N* = 10)**		**Corpus uteri (*N* = 14)**		**Ovary (*N* = 8)**	
	**at consultation**	**at consultation**	**at consultation**	**after consultation** ^1^	**at consultation**	**after consultation** ^1^	**at consultation**	**after consultation** ^1^	**at consultation**	**after consultation** ^1^	**at consultation**	**after consultation** ^1^	**at consultation**	**after consultation** ^1^
Patient sex (Male / Female)	231 / 280	92/42	23/7		13/3		56/0		0 / 10		0 / 14		0 / 8	
Patient age (Median / Mean / Range)	71 / 69 / 28–96	71/69/28–94	76/73/42–88		64/63/42–89		75/73/53–94		44 / 50 / 28–73		71 / 69 /55–82		64 / 68 / 48–87	
New < 180 days / New > 180 days / Recurrent	307/ 134/ 70	80/34/20	17/8/5		12/2/2		33/17/6		6 / 1 / 3		8 / 3 / 3		4 / 3 / 1	
**Abdominal symptoms**														
Abdominal pain, upper part	45	6			2	2	2		1				1	
Abdominal pain, lower part	37	8	1	1	2	1	3	1			1		1	
Constipation	22	3			1	1	2							
Diarrhoea	16	2				1					1		1	
Distended abdomen, bloating	27	4	1		1								2	3
Increased belching, flatulence	17	2	1		1									
Acid regurgitation	14	2			1		1							
Rectal bleeding	18	0												
Unexpected genital bleeding	4	4							1	1	3	7		
Haematuria, macroscopic ^2^	7	6	4	5	1	3	0	2			1	1		
Increased urinary frequency	14	6	2	2 ^*^			3	16 ^*^			1			
Other abdominal problems	34	5	1				3	1 ^**^		1 ^^^			1	
Only one abdominal symptom	66		4		2		8		2		2		3	
More than one abdominal symptom	63		2		2		3		0		2		1	
Any abdominal symptom	129 (25%)		6 (20%)		4 (25%)		11 (20%)		2 (20%)		4 (29%)		4 (50%)	
No symptom recorded	382		24		12		45		8		10		4	
**Non-specific symptoms (given at least one abdominal symptom)**														
Lack of appetite	26	3			1		1				1			
Unusual tiredness	25	6			2		1				2		1	
Involuntary weight loss	18	2	1			2							1	
Only one general symptom	24		1		1						1		2	
More than one general symptom	19				1		1				1			
Any general symptom	43 (33%)		1		2		1		0		2		2	

^1^ After consultation, i.e. between consultation and diagnosis: Symptoms not already recorded at consultation for these patients. "Only one..", "More than.." and "Any abdominal symtom" in this column count patients who had no symptom at consultation

The GPs' medical journal based free text comments are the basis for registration of symptoms after consultation. Only clearly stated symptoms are included in the table

"Abdominal pain" and "Acute abdomen" in free text have both been recorded as both upper and lower abdominal pain. "Changed bowel habit" recorded as both constipation and diarrhoea

^2^There were 10 more patients with bladder cancer and 3 more patients with kidney cancer and 1 more with prostate cancer who had haematuria, but where the GP did not specify whether it was macrocopic or microscopic. These are recorded with Microscopic haematuria in Table 2

^*^Different urininary symptoms of LUTS (Lower Urinary Tract Symptoms) type. These are supposed to include Increased urinary frequency

^**^Blood in sperm

^^^Vaginal discharge

### The most predominant abdominal symptoms recorded

‘Macroscopic haematuria’: Seven (1.4%) cancer patients in our study had this symptom at the initial consultation, of whom six patients with urogenital cancer. Four were subsequently diagnosed with bladder cancer, one patient with renal and one with uterine body cancer. An additional 13 patients with bladder cancer had haematuria before diagnosis, at least five of them macroscopic, meaning that more than half of the bladder cancer patients had macro- or microscopic haematuria before diagnosis. In the case of renal cancer, three patients had macroscopic haematuria after the initial consultation; two patients with prostate cancer also had a recording of macroscopic haematuria after the initial consultation. Macroscopic haematuria had been recorded for two patients who were subsequently diagnosed with uterine body cancer. In Tables 1 and 2, only macroscopic haematuria has been counted, except for groups C and D in Table 2.

**Table 2:** Sensitivity and specificity of 'Macroscopic haematuria' and 'Increased urinary frequency' according to cancer diagnosis, with corresponding positive predictive values of urogenital cancer

	N			Sensitivity (A)		PPV (A)				Sensitivity (B)		Sensitivity (C)		Sensitivity (D)	
	All	Males	Females	All	95% CI	All	95% CI	Males	Females	All	95% CI	All	95% CI	All	95% CI
**Bladder or renal cancer combined**	46	36	10												
Macroscopic haematuria and cancer (A):	5	4	1	10.9%	4.7–23.0	3.4%	1.5–7.7	4.9%	1.5%						
Macroscopic haematuria and cancer (B):	13	11	2							28.3%	17.3–42.6	56.5%	42.2–69.8	60.9%	46.5–73.6
**Bladder cancer**	30	23	7												
Macroscopic haematuria and cancer (A):	4	3	1	13.3%	5.3–29.7	2.7%	1.1–7.0	3.8%	1.5%						
Macroscopic haematuria and cancer (B):	9	7	2							30.0%	16.7–47.9	63.3%	45.5–78.1	66.7%	48.8–80.1
Increased urinary frequency (A)	2	1	1	6.7%	1.9–21.3	< 0.1%									
Increased urinary frequency (B)	4	3	1							13.3%	5.3–29.7				
**Renal cancer**	16	13	3												
Macroscopic haematuria and cancer (A)	1	1	0	6.3%	1.1–28.3	0.7%	0.1–3.8	1.3%							
Macroscopic haematuria and cancer (B)	4	4	0							25.0%	10.2–49.5	43.8%	23.1–66.8	50.0%	28.0–72.0
**Prostate cancer**		56													
Macroscopic haematuria and cancer (A)		0		-		-									
Macroscopic haematuria and cancer (B)		2								3.6%	1.0–12.1	5.4%	1.8–14.6		
Increased urinary frequency (A)		2		3.6%	1.0–12.1	< 0.1%									
Increased urinary frequency (B)		18								32.1%	21.4–45.2				
**Uterine body cancer**			14												
Macroscopic haematuria and cancer (A)			1	7.1%	1.3–31.5	0.7%	0.1–3.8		1.5%						
Macroscopic haematuria and cancer (B)			2							14.3%	4.0–40.0				
'Increased urinary frequency' and cancer (A)			1	7.1%	1.3–31.5				< 0.1%						
'Increased urinary frequency' and cancer (B)			2							14.3%	4.0–40.0				

A: Number of patients, sensitivity, specificity and positive predictive value (PPV) based on consultation recordings

B: Number of patients and sensitivity based on all recordings from consultation to diagnosis

C: Sensitivity based on all recordings of Macroscopic haematuria and/or microscopic haematuria

D: Sensitivity based on all recordings of Macroscopic haematuria plus microscopic haematuria and/or Anemia. For microscopic haematuria and 'Anemia', see below

Specificity of 'Macroscopic haematuria' = 99.8% (95% CI 99.7–99.8), based on 143 patients with 'Macroscopic haematuria' without cancer, 61,019 patients with no 'Macroscopic haematuria' and no cancer

Specificity of Increased urinary frequency = 98.8% (95% CI 98.7–98.9), based on 737 patients with 'Increased urinary frequency without cancer', 60,425 patients with no 'Increased urinary frequency' and no cancer

Microscopic haematuria: 10 patients with bladder cancer and 3 patients with renal cancer and 1 patient with prostate cancer had a positive test, performed on clinical grounds, but without any mention of 'Macroscopic haematuria' at or after consultation

Anemia': Recorded in 2 patients with bladder cancer and 2 patients with renal cancer and 0 patients with prostate cancer when there was no recording of 'Macroscopic haematuria' at or after consultation

Of these, 2 patients (1 bladder, 1 kidney) also had microscopic haematuria

For the calculation of specificity, all cancer patients reported by the GPs (*n* = 640) were excluded from the total number

Based on all recordings from consultation to diagnosis, the sensitivity of ‘Macroscopic haematuria’ to bladder and renal cancer combined was 28.3% (17.3–42.6). The specificity for ‘Macroscopic haematuria’ was 99.8% (99.7–99.8). A PPV of 3.4% (1.5–7.7) was noted for ‘Macroscopic haematuria’ and bladder or renal cancer combined. In males aged 55–74, PPV = 7.1% (2.5–19.0) for ‘Macroscopic haematuria’ and bladder cancer.

‘Increased urinary frequency’ was recorded at consultation for 14 cancer patients; two had bladder cancer, three had prostatic cancer, and one had uterine body cancer. GPs described LUTS (lower urinary tract symptoms), implying increased urinary frequency as recorded in Tables 1 and 2, but also related symptoms described with varying precision. In bladder cancer, one patient with haematuria and one more patient developed LUTS. In prostate cancer, 16 patients with no symptoms at consultation were reported having LUTS. One patient had haematospermia, a symptom considered innocent in younger patients.

For ‘Increased urinary frequency’, the sensitivity to bladder cancer was 13.3% (5.3–29.7), to prostatic cancer 32.1% (21.4–45.2), and to uterine body cancer 14.3% (4.0–40.0). The specificity for ‘Increased urinary frequency’ was 98.8% (98.7–98.9).

‘Unexpected genital bleeding’ was initially described for four cancer patients, three had cancer of the uterine body and one had cancer of the cervix. Another seven patients with uterine body cancer, and one more cervical cancer patient had this symptom before diagnosis.

Table 3 shows how the presence of cervical and uterine body cancers may be indicated by ‘Unexpected genital bleeding’ or ‘Increased urinary frequency’. The sensitivity of ‘Unexpected genital bleeding’ was 20.0% (4.8–44.8) to cervix and 71.4% (47.8–95.1) to uterine body cancer.

**Table 3:** Sensitivity and specificity of 'Unexpected genital bleeding' according to cancer diagnosis, with corresponding positive predictive values of cervical and uterine cancer

	N	Sensitivity (A)		Specificity		PPV		Sensitivity (B)	
	Females	All	95% CI		95% CI	All	95% CI	All	95% CI
Cervical cancer	10								
'Unexpected genital bleeding' and cancer (A):	1	10.0%	8.6–28,6			< 0.1%			
'Unexpected genital bleeding' and cancer (B):	2							20.0%	4.8–44.8
'Unexpected genital bleeding, without cancer: 195 patients									
No 'Unexpected genital bleeding, no cancer: 60,967 patients				99.7%	99.6–99.7				
Uterine body cancer	14								
'Unexpected genital bleeding' and cancer (A):	3	21.4%	< 0.1–42.9			1.5%	0.2–3.1		
'Unexpected genital bleeding' and cancer (B):	10							71.4%	47.8–95.1

A: Sensitivity, specificity and PPV based on consultation recordings

B: Sensitivity based on all recordings from consultation to diagnosis

‘Distended abdomen, bloating’ was recorded in 27 patients, two of these were among the eight patients with ovarian cancer, one had bladder and one renal cancer. A further three of the eight patients with ovarian cancer had this symptom after consultation. In ovarian cancer, the two patients with this symptom and two more patients told the GP their abdominal circumference had increased.

‘Abdominal pain’, upper and/or lower part, was recorded for 45 and 37 cancer patients, respectively. Relatively few were among the urogenital cancer patients: three patients with renal cancer, five with prostate cancer and four patients with female genital cancer. Two of the latter had ovarian cancer. GPs reported ‘abdominal pain’ in only four more patients after consultation but before diagnosis.

Other abdominal symptoms mentioned in Table 1 were rare and without discernible associations with cancer diagnoses. A few patients had non-specific symptoms (Table 1) recorded in addition to abdominal symptoms.

### The initiation of the diagnostic process

Most patients with urogenital cancer were symptomatic (82.1%) and were referred from general practice, most often by the reporting GP (Table 4). GPs’ case finding played a role in 10.7% of prostate cancer patients and screening in 30.0% of cervical cancer diagnoses. The tests involved in these cases seemed to have been taken without any preceding presenting and recorded symptom. In 11.2% of cases the diagnosis was made incidentally, without a clear cancer suspicion. A GP referred the patient in 83% of bladder cancers, 81% of renal cancers and 75% of prostate cancers. In 38% of cases, the diagnostic process was initiated during the initial consultation. Fast track, non-urgent referral was used for 18.7% of eventual diagnoses, most often for symptoms such as irregular bleeding or by a test result like an elevated PSA—although, in some cases by clinical findings or GP-perceived poor general health. Urgent referral (13.4%) usually was initiated based on unexpected bleeding or on clinical findings, or in several cases both.

**Table 4:** Type of referral to specialist care, if any, among patients with symptomatic or asymptomatic initiation of the diagnostic process of urological cancer

Location	**Symptomatic**				**Asymptomatic**	
	PC, ordinary referral	PC, fast track referral	PC, urgent referral	SC, no referral	Screening	Incidentally
**Bladder, ureter, urethra (30)**	12	7	5	3		3
Symptomatic 27, Asymptomatic 3						
** Renal Cancer (16)**	8	1	3	2		2
Symptomatic 14, Asymptomatic 2						
** Prostate (56)**	26	12	1	2	6	9
Symptomatic 41, Asymptomatic 15						
**Cervix uteri (10)**						
Symptomatic 7, Asymptomatic 3		2	2	3	3	
**Corpus uteri (14)**						
Symptomatic 14, Asymptomatic 0	7	3	3	1		
**Ovary (8)**						
Symptomatic 7, Asymptomatic 1	3		4			1

Reasons for urgent referral:

Bladder: Macro haematuria in 2 patients at consultation and 1 after consultation. 2 patients with lumps (neck, supraclavicular)

Renal: Macro haematuria in 1 patient at consultation and 1 after consultation. 1 patient with deteriorating general condition and anemia

Prostate: Urinary retention at consultion: 1

Cervix: 1 patient with Unexpected genital bleeding at consultation. 1 patient with Hemoptysis (recurrent cancer)

Corpus: At consultation: 2 patients with Unexpected genital bleeding, postmenopausal, and 1 patient with Macro haematuria and Increased urinary freqency

Ovary: At consultation: 2 patients with Distended abdomen, bloating, and also increased abdominal circumference. One of them also had upper abdominal pain, diarrhoea, fatigue, and a palpable tumour

2 patients without pre-recorded abdominal symptoms consulted for increased abdominal circumference. One of these also had a palpable tumour

Reasons for Fast track referral:

Bladder: Macro hamaturia in 2 patients at consultation and 3 patients after consultation. 1 patient had increasing abdominal pain and 1 had a deteriorating general condition (recurrent cancer)

Renal: 1 patient with Weight loss and anemia, at consultation

Prostate: Increased urinary frequency in combination with positive findings on digital examination: 1 patient at consultation and 2 patients after consultation. 1 patient with Increased urinary frequency and scrotal pain

2 patients had high PSA + digital examination findings. 1 had digital examination findings. 1 patient had Macro haematuria after first consultation, and 3 patients had increasing PSA. 1 unclear reasons for fast track

Cervix: 1 patient had vaginal discharge with cervical tumour on gynecological examination. 1 patient evoke GP's clinical suspicion when performing routine cervical smear

Corpus: 3 patients had Unexpected genital bleeding after first consultation

*PC* Primary Care, *SC* Specialist care/hospital

### The seriousness of disease

Slightly more than half of the cases were described as localised, 72 of 134. Clinical status was unknown for nine patients. For those with localised disease, 86.1% of the patients were feeling well or had stable disease on follow-up. In non-localised disease, this figure was 66.7%. Half of the cases of renal, uterine cervix and ovarian cancer were not localised (Table 5). These cancer types tended to have a lower percentage of patients feeling well or with stable disease. Overall, 22 of 125 patients (17.6%) had progressive disease or were dead on follow-up. This means that GPs see most urogenital cancer patients at a time when meaningful therapeutic action is possible. After fast-track referral, four of 25 patients (16.0%) had progressive disease or were dead on follow-up, while the figures for urgent referral were six of 17 patients (35.3%), with one unknown status.

**Table 5:** The clinical status of disease by stage and type of referral among patients with urogenital cancer

Type of cancer, with location	Clinical status at follow-up			Status after Fast track			Status after Urgent referral		
	Feeling well or stable disease	Progressive disease or dead	Unknown	Feeling well or stable disease	Progressive disease or dead	Unknown	Feeling well or stable disease	Progressive disease or dead	Unknown
**Bladder** (*N* = 30)									
Localised: 16 (53%)	14 (88%)	1 (6%)	1	3	1		1		1
Not localised: 11 (37%)	9 (82%)	2 (18%)		2	1		2		
Unknown: 3	1	2						1	
**Kidney** (*N* = 16)									
Localised: 7 (44%)	5 (71%)	0	2		1		1	2	
Not localised: 8 (50%)	3 (38%)	5 (63%)							
Unknown	1								
**Prostate** (*N* = 56)									
Localised: 31 (55%)	26 (84%)	1 (3%)		5					
Not localised: 18(32%)	14 (78%)	4 (22%)		6				1	
Unknown 7	5	1	1	1					
**Cervix uteri** (*N* = 10)									
Localised: 4 (40%)	4 (100%)	0		1					
Not localised: 5 (50%)	3 (60%)	2 (40%)			1		1	1	
Unknown: 1			1						
**Corpus uteri** (*N* = 14)									
Localised: 10 (71%)	9 (90%)	1 (10%)		3			3		
Not localised: 2 (14%)	2 (100%)	0							
Unknown: 2	2								
**Ovary** (*N* = 8)									
Localised: 4 (50%)	4 (100%)	0					3		
Not localised: 4 (50%)	1 (25%)	3 (75%)						1	

### The diagnostic role of the clinical examination, laboratory tests and diagnostic procedures ordered by the GP

Table 6 shows which diagnostic elements had an impact on the cancer diagnosis in different patients.

**Table 6:** The number of patients where clinical examination, laboratory tests and diagnostic procedures performed or ordered by a GP had diagnostic importance, in total and with symptomatic initiation

			**Cancer site**			
	**Bladder, ureter, urethra**	**Renal**	**Prostate**	**Cervix uteri**	**Corpus uteri**	**Ovary**
**Clinical examination**						
Abdominal examination	3 (3)	1 (1)	1 (1)		1 (1)	5 (5)
Digital rectal examination	1 (1)	2 (2)	27 (23)			
Gynecological examination	1 (1)			5 (3)	5 (5)	1 (1)
Proctoscopy/sigmoidoscopy						
Other examination, not specified	9 (8)	4 (4)	5 (4)		2 (2)	
No contribution from clinical examination	16 (14)	9 (7)	20 (11)	3 (2)	6 (6)	3 (2)
Missing	3 (3)	1 (1)	4 (3)	1 (1)		
**Laboratory tests**						
Haemoglobin concentration	2 (1)	2 (2)	2 (2)		1 (1)	
Erythrocyte Sedimentation rate	1 (0)	2 (2)	1 (1)		1 (0)	
C-Reactive Protein	1 (0)	1 (1)	1 (1)			1 (1)
Test for occult blood in stool	1 (1)	1 (3)				
Cervical cytology				3 (1)	1 (1)	
Prostate Specific Antigen			45 (32)			
Urinary examination	14 (14)	8 (7)	1 (1)		1 (1)	
Other	4 (3)	3 (3)	1 (1)	1 (0)	1 (1)	1 (1)^^
No diagnostic contribution from laboratory tests	10 (9)	5 (4)	6 (4)	3 (3)	9 (9)	5 (4)
Missing	2 (2)		4 (4)	2 (2)	1 (1)	1 (1)
**Diagnostic procedures**						
X-ray		1 (1)	3 (2)	1 (1)		
Ultrasound	7 (7)	2 (2)	10 (5)		4 (4)	3 (3)
Computer tomography	7 (5)	13 (11)	5 (4)	1 (1)	4 (4)	5 (4)
Magnetic resonance		3 (3)	8 (5)	1 (1)	1 (1)	
Upper GI Endoscopy	1 (1)					
Colonscopy	1 (1)					
Cystoscopy	21 (18)	3 (3)	5 (4)		1 (1)	
Other			13 (7)^*^	3 (1)^**^	3 (3)^	
None of the above	1 (1)		16 (12)	4 (3)	2 (2)	1 (0)
Missing	2 (2)		11 (10)	2 (2)	2 (2)	1 (1)
**Number of patients**	**30**	**16**	**56**	**10**	**14**	**8**

More than one examination/procedure could be recorded for one patient, where appropriate

Number of patients with symptomatic initiation of the diagnostic process in parentheses

^*^9 of these were biopsies of prostate

^**^Kolposcopy

^^^Cytology / histology of endometrium

^^^^CEA

In bladder cancer, the GP’s clinical examination played a modest role in diagnosis. Eleven of 30 patients had some diagnostic contribution from abdominal, gynaecological, rectal or unspecified examination, while clinical examination was said to give no contribution for 16 patients. Laboratory tests were more important, especially urinary testing, which contributed for 14 patients. Haemoglobin examination was noted for two patients. Laboratory tests did not contribute for ten patients. Diagnostic procedures mostly were performed in secondary care after referral and were important for all but one patient. Twenty-one patients had cystoscopy findings, seven ultrasound, and six CT findings.

In renal cancer, clinical examination also played a modest role with abdominal, digital or ‘other’ examinations reported in six patients. No contribution was reported for nine patients. Laboratory tests results influenced the diagnostic pathway in all but five patients. Urinary examination was useful for eight patients, and a low haemoglobin concentration was important in two cases. Diagnostic procedures were important for all, mainly CT for 13 patients.

In prostate cancer, clinical examination contributed in 32 (57%) of the 56 patients, mostly digital rectal examination. There was no contribution from clinical examination in 20 patients. PSA was the dominant laboratory test and contributed to diagnosis in 45 (80%) patients. Laboratory tests did not contribute for six patients. Diagnostic procedures contributed to the diagnosis of 29 patients.

For the three female cancers, abdominal and gynaecological examinations were important parts of the diagnostic procedure. For the few patients with ovarian cancer, abdominal examination played a greater role than what was suggested by the symptom recordings at consultation. Cervical cytology was the only single laboratory test that played an important role. For uterine body and ovarian cancer, ultrasound and CT were important procedures.

### Country differences

The country-specific distribution of different types of cancer was not significantly different from the distribution of patients. Fast track referral was used in 30% of Danish patients, while this figure was 16% for the other countries combined. The difference is not significant. For prostate cancer, digital rectal examination was reported diagnostically useful for about half of the patients, but for none of eight patients from Belgium. In Scotland, there were no patients with prostate cancer. The diagnostic contribution from PSA was similar in the five other countries. Other differences in diagnostic pathway influence of testing were small. There were no significant differences in the patients’ disease stage and clinical state at follow-up.

## Discussion

### Main findings: the symptoms and the cancers

The study findings suggested that ‘Macroscopic haematuria’ was the most important single symptom associated with bladder cancer, and that it may also signal renal cancer or prostate cancer. Several cases were clarified through the GP’s clinical examination, or laboratory investigations. The novelty of this study is that symptoms related to urogenital cancers have been studied prospectively in a random primary care population in six countries.

For each of the urogenital cancers included in our study, at least one presenting symptom was both sensitive and specific enough to merit an intentional GP investigation. As in all cancer, vague symptoms or low-risk-but-not-no-risk symptoms [19] are sometimes the GP’s only initial clue to diagnosis. However, this problem is perhaps less important in urogenital than in colorectal cancer, where all kinds of abdominal symptoms may be the presenting symptom [3]. The lower rate of localised disease in renal and uterine cervix and ovarian cancer could encourage GPs to put more weight on atypical symptoms if the GP intuition suggests one of these cancers [1, 20]. The relatively low proportion of urogenital patients who experienced progressive disease or death within the time frame of our study, means that GPs see most urogenital cancer patients at a time when meaningful therapeutic action is possible.

When considering microscopic haematuria and/or anaemia as other possible signs of cancer in the urinary tract, sensitivity of at least one of these signs increased to two of three for bladder cancer and one of two for renal cancer.

The other three symptoms with high sensitivity were mainly related to one type of cancer: ‘Increased urinary frequency’ to prostate cancer, ‘Unexpected genital bleeding’ to uterine body cancer, ‘Distended abdomen, bloating’ to ovarian cancer. ‘Abdominal pain’, both upper and lower, rarely were recorded in the urogenital cancer patients, but it may be noted that abdominal pain occurred in the otherwise relatively symptom-poor renal and ovarian cancers. Although abdominal pain is a rather a-specific symptom, it is important to include these cancers in the differential diagnosis in the absence of another explanation.

Prostate cancer is frequently a relatively symptom-poor or asymptomatic cancer. However, only one quarter of cases were asymptomatic and more of these were diagnosed incidentally than by PSA testing/case finding; screening is not being recommended in countries participating in this study. ‘Increased urinary frequency’ mainly occurred as part of LUTS, which is frequent in elderly men with benign prostatic hyperplasia (BPH), and thus a symptom with low specificity in relation to cancer. In fact, BPH is not a risk factor for prostate cancer [21]. Despite this, LUTS symptoms should be acknowledged as a symptom where a case-finding approach toward prostate cancer should be weighed against the burden of invasive investigations and uncertainty attached to treatment approaches. After a rectal examination, these issues should be discussed with the patient. Further investigation, often starting with a PSA or another chemical screening test, should be considered if this becomes the patient’s informed choice. One ‘prostate paradox’ is shown here, in that digital rectal examination was important for almost half of the patients. An ‘early’ T1 cancer is generally not detectable by palpation, but this does not make a digital rectal examination worthless. A positive GP rectal examination represents an opportunity for referral and a specialist examination and evaluation of treatment possibilities if cancer is diagnosed.

Increased urinary frequency was otherwise rare in urogenital cancer but occurred sporadically in bladder cancer and uterine body cancer.

In relation to uterine cancer, ‘Unexpected genital bleeding’ was confirmed as a predominant symptom in uterine body cancer and an important symptom in cervical cancer. A specificity of 99.7% in relation to cancer underlines the importance of always finding an explanation when such bleeding occurs in postmenopausal and generally also in middle-aged and younger women even when positive predictive values are low.

There were only eight cases of ovarian cancer, but five of them had a recording of ‘Distended abdomen, bloating’, confirming previous studies suggesting that this is a symptom to be taken seriously in women. Analysis of reasons for urgent referral in four of these patients suggests that GPs should both measure abdominal circumference and ask about possible change in patients with this symptom, and that an abdominal palpation in some cases may reveal a palpable tumour.

Our study confirms the major role of the GP in initiating diagnostic procedures [22]. The study contributes to understanding which clinical examinations, laboratory tests and supplementary procedures are the most important for diagnostic urogenital work-up in general practice.

Country differences were small, probably because the encounter between patient and GP is rather similar in the participating countries.

### Discussion within the context of international literature

It has been shown previously that abdominal symptoms commonly precede various diagnoses of abdominal cancer [16]. PPV is the chance of a patient having the disease of interest when they have reported the symptom [23]. Our PPVs are in line with figures in other studies [24]. We agree with the NICE recommendation to consider 3% as a reasonable threshold for referral [25], based on symptoms and other information the GP can obtain. For the non-specific symptoms studied here, it has previously been shown that they have low cancer relevance in themselves, but that they gain in importance when associated with an alarm symptom [14].

Macroscopic haematuria is an important symptom in that it dominates the pre-diagnostic symptoms of bladder cancer [26, 27], although the non-specific features of dysuria, malodorous urine, urge and urinary retention have been described as well [16, 28, 29]. Delay in bladder cancer has been reported more important in women [28]. There were only a few female patients in our study, but they had symptoms similar to males.

A meta-analysis found PPV 5.1% for visible haematuria in relation to bladder or renal tract cancer [10]. This is slightly higher than our figure (Table 2). Renal cancer may be symptom-poor, but haematuria occurred in our study and is not uncommon. One article says that 30% of renal cancer patients are diagnosed on the basis of symptoms [30]. Another primary care study found 57% [16]. Macroscopic haematuria is not specific for cancer [1] but appears to be a rare symptom in non-urinary types of cancer. A fallacy here is that patients and GPs may perceive an unexpected genital bleeding to be haematuria, exemplified in our study. Men over 60 years with haematuria had the highest PPV for urological cancer in a Belgian-Dutch study [31]. Our highest values for males were in the age group 55–74 years. Due to the limited number of cancer patients, computing other age specific PPVs was not feasible in our study.

For bladder and renal cancer, it has been shown that abnormalities in blood tests may also signal early cancer [32], confirming the importance of laboratory tests in many of our patients.

LUTS may signal prostatic disease, but not whether it is benign or malignant. However, the symptom had high sensitivity for cancer in our study. This is in accordance with a previous study from Norwegian general practice [16]. One patient with haematospermia is in line with the acknowledged rare occurrence of this symptom in cancer of the prostate [33]. In articles from urological groups, the emphasis is commonly on PSA and newer diagnostic markers rather than on symptoms [34].

Several clinical features are associated with uterine cancer, and unexpected genital bleeding of different kinds are most important [35–37]. The kind of ‘Unexpected genital bleeding’ most frequently associated with cancer is postmenopausal bleeding. Among more than 10,000 patients with a first consultation for postmenopausal bleeding in British general practice, 1.7% received a relevant cancer diagnosis within two years [38]. Data from guideline working groups give much higher figures, i.e. about 20% of patients with postmenopausal bleeding are diagnosed with a malignancy, endometrial most commonly, but also cervical, quoted in [39]. This may suggest how important it is to have data collected both in general practice and in hospitals. Our 14% Fig. (12 of 13 patients in Table 2 were 55 + years of age) is on the higher side. In younger women, cervical cancer is rare although possible, and post-coital bleeding and intermenstrual bleeding should lead to visualization of the cervix and smear examination [40]. Vaginal discharge triggered visualization of cervix and a cancer diagnosis in one of our patients. In a case–control study using electronic records from primary care, postmenopausal bleeding, excessive vaginal bleeding and irregular menstruation were the main features associated with uterine cancer [35].

‘Distended abdomen, bloating’ has been described as potentially important for earlier diagnosis of the reputed “symptom-poor” ovarian cancer [41, 42]. In our study with only eight cases of ovarian cancer, five had ‘Distended abdomen, bloating’. This high sensitivity contrasts with the low PPV for women; 1.9% [1], abdominal distention being a common symptom for many reasons. Symptoms of ovarian cancer may not be well-recognised by women in the general population [43], and this includes abdominal distention. Direct referral access to transvaginal ultrasound when a GP suspects ovarian cancer has proved feasible and useful [44].

Patients with fast-track referral did not fare better than patients with other types of referral in this study. A study from 2013 [45] suggests that cutting down on long diagnostic intervals may lead to better survival. Standardized cancer patient pathways shorten time between consultation and treatment and offer a meaningful approach when the total information increases the possibility of cancer [46, 47]. Recently, a grounded theory study from a primary care perspective explored how cancer could be diagnosed in a more timely way. This study pointed at pluralistic task shifting including primary care tasks like cognitive tasks and digital tasks to achieve this [48]. For all cancers, it should be kept in mind that delays between diagnosis and surgery still seems to be associated with a relative increase in all-cause mortality [49].

### Strength and limitations of the study

There were few patients for each of the six types of urogenital cancer, limiting to some extent the generalizability of findings. However, this is compensated by the detailed information throughout the diagnostic cycle for each patient, and by the prospective nature of the follow-up. The UK NICE guidelines use a 3% risk threshold for recommending a suspected cancer pathway referral [25]. Based on Bayesian thinking, combinations of symptoms and signs may bring the cumulative PPV above 3% [10, 30]. Neither the patient nor the GP knew about the cancer at the time of the initial consultation. It cannot be excluded that symptoms have been presented before, as all the first 20 consecutive consultations per day with patients 16 years of age and older were registered, regardless of previous symptom presentations. Data from medical records can be incomplete, but these kind of symptom data probably have high reliability [50]. Consecutive patients were registered sequentially, with no selection bias. The initial registrations were carried out in the GP’s surgery, which is the setting where the real-life diagnostic considerations are performed. The patient form was simple, with multiple choice answers and room for free text comments. Whether the GPs were representative for their profession was considered unimportant because new cancer patients are haphazardly distributed among GPs.

The registration form included an option to tick off for the statement “Symptoms suggest cancer”, which might be useful to consider in the assessment of the diagnostic role of clinical examinations, laboratory tests and diagnostic procedures. Due to missing values in 77% of the cancer patients, the information provided was too limited to be applied.

With time, it becomes gradually less probable that there is a relationship between symptom and cancer. In the second article from this study [2], we limited analysis to patients diagnosed within six months after the consultation. However, there is no sharp cut-off at six months, rather it was the limit that seemed reasonable when planning the study. With few patients in each diagnostic group, we chose to include all patients in the study and assume a relationship between the symptom and cancer. Sensitivity analyses with comparison of all 511 cancer patients with patients diagnosed within six months have suggested that symptoms recorded > 180 days before diagnosis may be less related to subsequent cancer, but the difference is not great [2]. We therefore think our assumption is true in most cases. For example, there are probably few patients with bladder cancer where a macroscopic haematuria is not related to the cancer at the time of diagnosis.

## Implications for policy, practice and research

GPs routinely encounter patients where a urological cancer can be suspected. The GP has an important initial role in recognising that a particular patient may have such a cancer. The diagnostic search should ideally exclude or confirm the relevant form of cancer or lead to a referral if ambiguity persists. On the other hand, other symptoms were rarely specific for these types of cancer. Our study shows the importance of recognising symptoms that are sensitive in relation to individual types of urogenital cancer, and of exploring further the probability of cancer. Most GPs will react to red flag symptoms like postmenopausal bleeding and macroscopic haematuria in elderly persons, while a symptom like abdominal distention requires more attentive listening. Critically, our study highlights the importance of relevant, conventional examinations the GP can perform to elucidate the importance of the symptoms. It clarifies the interplay between symptom, clinical findings and supplementary tests and examinations. Such knowledge should continue to be developed in a collaborative manner and in parallel between primary and secondary care. We believe that large primary care multi-centre studies in the future could improve our understanding about how to approach specific types of cancer.

## Data Availability

The dataset analysed during the current study is available in the UiT Open Research Data repository.
